# Exploring the Efficacy of Physiotherapy in Guillain-Barré Syndrome Through Virtual Reality-Based Rehabilitation: A Case Report

**DOI:** 10.7759/cureus.59042

**Published:** 2024-04-26

**Authors:** Neha P Arya, Nikita H Seth, Raghumahanti Raghuveer, Yogesh Sewani

**Affiliations:** 1 Neurophysiotherapy, Ravi Nair Physiotherapy College, Datta Meghe Institute of Higher Education and Research, Wardha, IND; 2 General Medicine, Indira Gandhi Government Medical College and Hospital, Nagpur, IND

**Keywords:** case report, balance, physiotherapy, rehabilitation, virtual reality, guillain barre syndrome

## Abstract

Guillain-Barré syndrome (GBS) refers to a spectrum of acute immune-mediated polyradiculoneuropathies, among which is acute motor axonal neuropathy (AMAN), which is typified by predominant motor involvement and axonal degeneration. This case study describes the presentation, diagnosis, and physiotherapy management using virtual reality-based technology in a 29-year-old male patient with AMAN. Nerve conduction velocity testing was used to diagnose motor axonal neuropathy in the patient, who had weakness subsequent to gastrointestinal symptoms. Intravenous immunoglobulin therapy was started, and a physiotherapy protocol was planned for eight weeks according to the patient’s functional status. Physiotherapy plays an important role in the rehabilitation of patients with GBS, addressing the specific motor deficits and promoting recovery. The aim was to improve muscle strength, mobility, and functional independence through progressive exercises targeting specific motor deficits. Virtual reality-based training was also part of this rehabilitation process as an adjunct to conventional rehabilitation to improve dynamic balance and function of the upper and lower limbs, which showed significant improvement in the outcome measures.

## Introduction

Guillain-Barrè syndrome (GBS) is an acute immune-mediated polyradiculoneuropathy caused by inflammation of the peripheral nerves and nerve roots. It stands as the leading cause of acute paralysis linked to neuropathy [1,2]. A significant proportion of cases, around 50-70%, manifest approximately one to two weeks following a respiratory or gastrointestinal infection or some other immune-triggering event [3]. In 2019, there were 150,095 total cases of GBS worldwide. Globally, there was a 6.4% increase in the age-standardized prevalence of GBS per 100,000 population between 1990 and 2019 [4]. These occurrences incite an irregular autoimmune reaction targeting the peripheral nerves and their spinal roots. It is a multifaceted and diverse syndrome brought on by different kinds of lesions [5,6]. While acute axonal motor neuropathy (AMAN) is less frequent, featuring predominantly axonal injury and exclusive motor impairment, acute motor-sensory axonal polyneuropathy (AMSAN) shares a similar pathogenesis with AMAN but includes sensory deficits [7]. The most prevalent form is acute inflammatory demyelinating polyradiculoneuropathy (AIDP), characterized primarily by demyelinating features. Studies have shown that utilizing virtual reality (VR) for rehabilitation can enhance both motor and cognitive abilities across diverse patient groups [8,9]. Using a combination of visual, aural, tactile, and somatosensory stimuli, VR is a type of digital treatment that allows users to train in a setting that closely resembles actual objects and events [10]. In addition to improving outcomes like postural balance, quality of life, and perceived confidence in balance, it encourages patients to engage in rehabilitation [11].

Physiotherapy is vital for the treatment of GBS and all of its variants, including AMAN. Considering its critical function, there is still a lack of research on the particular rehabilitation approaches designed for AMAN. Thus, it becomes essential to record customized physiotherapy regimens and their results in such instances in order to inform evidence-based practice [12,13]. This case study adds to the increasing database of research on rehabilitation techniques by clarifying the particular physiotherapy interventions used and their effect on the patient's recovery course. Moreover, it highlights the importance of timely identification, appropriate treatment initiation, and customized rehabilitation regimens in maximizing results for those impacted by this form of GBS.

## Case presentation

A 29-year-old male presented with difficulty lifting both arms, walking, and swallowing. He reported a history of abdominal pain, diarrhea, and vomiting for the past five days, for which treatment was done by a local practitioner. Two days ago, he experienced the inability to lift his shoulder, which progressed to weakness in the distal muscles of his forearm and hand the next day, along with weakness in both his lower limbs. The patient had a history of pulmonary tuberculosis three years ago and was treated for six months. A clinical examination was performed, and the patient was referred for investigation. A magnetic resonance imaging (MRI) of the brain, cervical, and lumbar spine was normal. Nerve conduction velocity (NCV) testing revealed motor axonal polyneuropathy. The patient was admitted to the neurology intensive care unit (ICU) and received immunoglobulin (total: 150 gm) therapy in a total of 25 cycles along with that Tab. Neurobion Forte was given for 15 days. He also experienced breathing difficulty but maintained SpO2 levels. The patient was referred for neurophysiotherapy and was later discharged from the hospital. He continued physiotherapy rehabilitation at the outpatient department.

Clinical findings

Informed consent was taken before the examination. The patient was conscious and oriented to time, place, and person. He was hemodynamically stable. A detailed neurological assessment was taken. Hypotonia was found in the bilateral upper and lower extremities according to the Tone Grading Scale (TGS). Deep tendon reflexes were absent. All superficial, deep, and combined cortical sensations were intact. Manual Muscle Testing (MMT) was done at the end of the second week after discharge from ICU, which showed reduced muscle strength for bilateral upper and lower limbs, grade 3+ (complete range of motion against gravity). Examinations for other outcome measures were also done, including the Berg Balance Scale (BBS), Huges disability index for GBS, and Functional Independence Measure (FIM). The patient's height was 182 cm, and weight was 67 kg.

Investigations

MRI of the brain and complete spine was done upon the patient’s arrival at the hospital, which did not reveal any abnormality. NCV testing was done, which showed motor axonal neuropathy. Compound muscle action potential (CMAP) could not be elicited in bilateral ulnar, tibial, and peroneal nerves. CMAP and distal motor latency and conduction velocity were within normal limits in bilateral median nerves. Sensory nerve action potential (SNAP) amplitude is within the normal limit in bilateral upper and lower limbs. Table 1 depicts motor NCV findings.

**Table 1:** Motor nerve conduction velocity findings mv: millivolts; ms: milliseconds; m/s: meter per second

Nerve	Amplitude	Duration	Conduction velocity	Conduction velocity (normal range)
Right median	0.7 mv	12.3 ms	50.6 m/s	50-70 m/s
Left median	4.8 mv (wrist); 0.6 mv (axilla)	10.2 ms (wrist); 6.15 ms (axilla)	76 m/s	50-70 m/s
Right ulnar	0 mv	0 ms	0 m/s	50-70 m/s
Left ulnar	0 mv	0 ms	0 m/s	50-70 m/s
Right peroneal	0 mv	0 ms	0 m/s	40-60 m/s
Left peroneal	0 mv	0 ms	0 m/s	40-60 m/s
Right tibial	0 mv	0 ms	0 m/s	40-60 m/s
Left tibial	0 mv	0 ms	0 m/s	40-60 m/s

Physiotherapy management

After a detailed examination, the physiotherapy protocol was planned for eight weeks. The patient was admitted to ICU until the second week. Further rehabilitation was planned in the outpatient department. Progression was made in the exercises according to the patient's functional capacity. Table 2 shows physiotherapy management for weeks 1 and 2.

**Table 2:** Physiotherapy protocol for weeks 1 and 2 PNF: Proprioceptive Neuromuscular Facilitation

Goals	Intervention	Repetitions
To improve inspiratory capacity	Incentive spirometry	10 reps × 1 set (2-3 times/day)
To improve functional mobility and prevent bed sores	Bed mobility exercises	10 reps × 1 set
To facilitate muscle contraction	Facilitatory approaches: Quick icing, joint approximation, quick stretch, tapping	5 reps × 1 set
To initiate movement and improve muscle tone	PNF rhythmic initiation, D1 flexion-extension for upper limb, D2 flexion-extension for lower limb	10 reps × 2 sets

Table 3 depicts the physiotherapy protocol for weeks 3-5.

**Table 3:** Physiotherapy protocol for weeks 3-5 LE: lower extremity; SLR: straight leg raise; UE: upper extremity; VR: virtual reality; VO_2_max: maximal oxygen consumption

Goals	Intervention	Repetitions
To improve muscle strength of the upper limb	Strengthening exercises using a 1-kg dumbbell (week 3), progressing to a 2- to 3-kg dumbbell (weeks 4 and 5). Shoulder flexors and extensors, abductors and rotators, bicep curls, triceps strengthening, wrist strengthening, gripping activities (putty, sponge ball).	10 reps × two sets
To improve core and back strength	Abdominal curls in supine with arms aside (week 3), progressing to abdominal curls, keeping hands at the back of the head (weeks 3 and 4). Prone on the forearm (week 3), progressing to prone on hands (weeks 4 and 5). Pelvic bridging exercise.	10 reps × two sets
To improve scapular stability	Prone arm flexion (weeks 3 and 4). Prone T exercise with bilateral arms and thumbs up (weeks 3 and 4). Prone rowing with 1-kg dumbbell (weeks 4 and 5). Serratus wall slides (week 3) progressed with wall slides with resistance band (weeks 4 and 5).	10 reps × one set
To improve muscle strength of the lower limbs	Strengthening exercises with 1-kg weight cuff (week 3) progressing to 2-kg weight cuff (weeks 4 and 5), SLR, hip abduction in side lying, prone hip knee extension, dynamic quadriceps, and hamstring curls.	10 reps × two sets
To train functional activities	Kneel sitting to kneeling, kneeling to half kneeling, half kneeling to standing.	5 reps × one set
To improve co-contraction and joint stability	Closed kinetic chain exercises for lower extremities. Wall-supported mini-squats (weeks 3 and 4) progressing to unsupported mini-squats, side lunge, and front lunge (weeks 3-5).	10 reps × one set
To improve trunk and pelvis stability	Rhythmic stabilization for trunk and pelvic muscles.	10 reps × one set
To improve cardiovascular endurance	Treadmill walking.	Three times/week 30-40% VO_2_ max progressing to five times/week 50-70% of VO_2_ max
To improve static and dynamic balance	One leg standing with eyes open, progressing to standing with eyes closed. Perturbation-based balance training started with small amplitude perturbations and progressed to medium frequency perturbations. Balancing activity on the wobble board. Balance while standing while resistance is provided to the arms via a resistance band.	15 reps × one set
To improve UE and LE function and balance	VR-based training.	VR training: 20 mins

**Figure 1:**
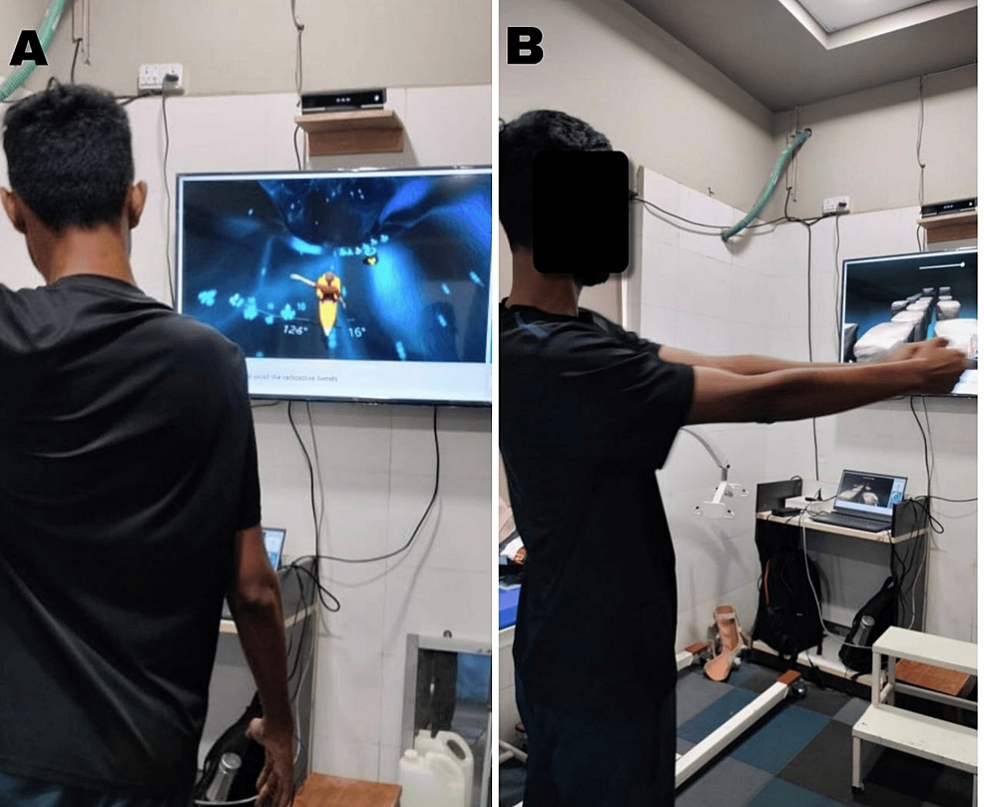
Patient performing virtual reality-based training A: Patient performing trunk activity. B: Patient performing upper limb activity.

Figure 1 shows the patient performing VR-based training.

**Figure 2:**
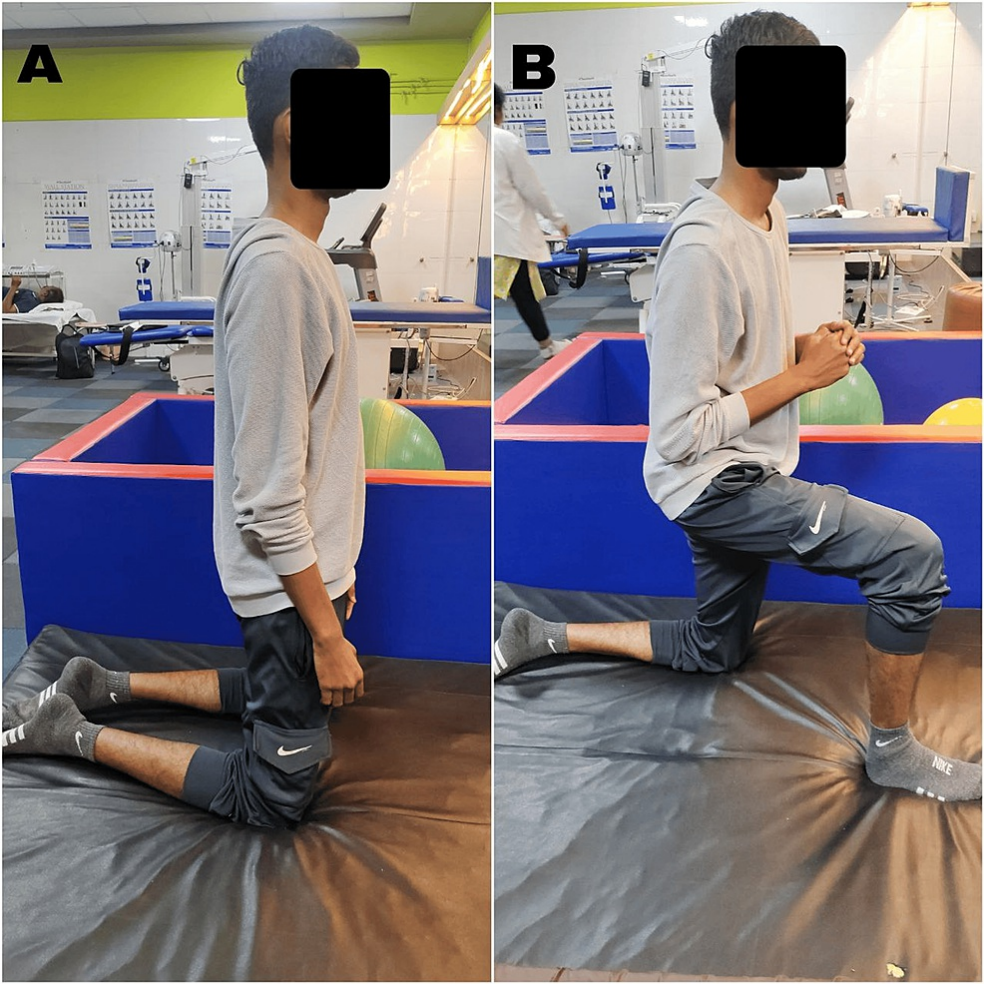
Patient performing transition from kneeling to half-kneeling A: Patient performing kneeling. B: Patient performing half-kneeling.

Figure 2 depicts the patient performing a transition from kneeling to half-kneeling.

Follow-up and outcome measure

Outcome measures were examined at the beginning of the rehabilitation at the outpatient department. Significant improvement was observed in muscle strength, balance, and independence in activities of daily living, as well as the Huges disability scale for GBS at the end of the rehabilitation program. Table 4 shows assessment findings for MMT (reliability: 18).

**Table 4:** MMT findings for upper and lower limbs MMT: Manual Muscle Testing

Muscle group	Action	Week 2		Week 8	
		Right	Left	Right	Left
Shoulder	Flexors	3+	3+	5+	5+
	Extensors	3+	3+	5+	5+
	Abductors	3+	3+	5+	5+
	Adductors	3+	3+	5+	5+
	Internal rotators	3+	3+	5+	5+
	External rotators	3+	3+	5+	5+
Elbow	Flexors	3+	3+	5+	5+
	Extensors	3+	3+	5+	5+
Wrist	Flexors	3+	3+	5+	5+
	Extensors	3+	3+	5+	5+
Hip	Abductors	3+	3+	5+	5+
	Adductors	3+	3+	5+	5+
	Flexors	3+	3+	5+	5+
	Extensors	3+	3+	5+	5+
Knee	Extensors	3+	3+	5+	5+
	Flexors	3+	3+	5+	5+
Ankle	Plantar flexors	3+	3+	5+	5+
	Dorsi flexors	3+	3+	5+	5+

Table 5 depicts the strength assessment for trunk muscles.

**Table 5:** Strength assessment for trunk muscles

Muscle	Week 2	Week 8	Week 10 (Follow-up)
Upper abdominal muscles (trunk flexors)	Grade 2	Grade 5	Grade 5
Trunk rotators	Grade 2	Grade 5	Grade 5
Trunk extensors	Grade 2	Grade 5	Grade 5
Side bridge endurance test	Four seconds	24 seconds	26 seconds

Table 6 shows examination findings for outcome measures.

**Table 6:** Examination findings for outcome measures pre- and post-intervention GBS: Guillain-Barré syndrome

Outcome measure	Week 2	Week 8	Week 10 (Follow-up)
Berg Balance Scale	42/56	56/56	56/56
Huges disability scale for GBS	3	1	0
Functional Independence Measure	112/126	126/126	126/126

## Discussion

The term GBS refers to a group of acute inflammatory polyneuropathies, of which there are different subtypes, each with unique pathological and clinical characteristics. It has been demonstrated that using VR as a rehabilitation tool can improve motor and cognitive function in a variety of populations [14]. Hao Feng et al. did a study on patients with Parkinson's disease to compare the effects of VR-based rehabilitation to conventional rehabilitation in improving balance and gait and found that 12 weeks of VR-based rehabilitation gives better results than conventional rehabilitation in improving balance and gait [15]. Jiayin Chen et al. analyzed 42 trials to find the effectiveness of VR-based exercise therapy for upper extremity motor rehabilitation in stroke patients and found that VR is effective in upper extremity motor function [16,17]. Kate E Laver et al. conducted a review of 72 trials to assess the effectiveness of VR compared to other interventions or no intervention in improving upper limb function and activity [18-20]. Their findings suggest that VR and interactive video gaming did not show greater benefits than traditional therapy methods for enhancing upper limb function [21].

The patient, in this case, had acute AMAN, a distinct form of GBS characterized by axonal degeneration without a major demyelination and predominant motor involvement. The clinical presentation of the patient, characterized by the acute onset of descending paralysis involving both upper and lower extremities, aligns with the diagnosis of AMAN. The absence of sensory deficits and preserved sensory nerve conduction velocities on nerve conduction studies support the motor-predominant nature of the neuropathy characteristic of AMAN. Diagnostic evaluation, including MRI of the brain and spine, ruled out structural lesions, supporting the diagnosis of peripheral neuropathy. NCV testing revealed features consistent with motor axonal neuropathy, confirming the diagnosis. Management primarily involves supportive care and immunomodulatory therapy aimed at attenuating the immune-mediated attack on peripheral nerves.

In this case, the patient received intravenous immunoglobulin (IVIG) therapy, which has been shown to hasten recovery and improve outcomes in patients with GBS and its variants. Physiotherapy was important for the patient's recovery as it improved the patient's muscle strength, mobility, and functional independence. Over eight weeks, the systematic physiotherapy regimen was designed to treat the specific motor deficits associated with the condition. The interdisciplinary approach proved to be helpful in optimizing outcomes for individuals with GBS, as evidenced by the notable improvements found in various outcome measures such as muscle strength, balance, and functional independence.

## Conclusions

The study defines the treatment and recovery of a patient with AMAN, a subtype of GBS. We addressed particular motor deficits with a customized eight-week rehabilitation program to improve muscle strength, mobility, and functional independence. VR-based training was also part of rehabilitation. Significant improvements were observed across various outcome measures, including muscle strength, balance, and functional independence. This case study provides significant insights into the interdisciplinary management of GBS and AMAN, highlighting the significance of early detection, timely intervention, and tailored rehabilitation approaches. As the study includes a single case, it requires additional research and clinical documentation to improve rehabilitation procedures and outcomes for those with such neurological disorders.
